# Supervised and Dynamic Neuro-Fuzzy Systems to Classify Physiological Responses in Robot-Assisted Neurorehabilitation

**DOI:** 10.1371/journal.pone.0127777

**Published:** 2015-05-22

**Authors:** Luis D. Lledó, Francisco J. Badesa, Miguel Almonacid, José M. Cano-Izquierdo, José M. Sabater-Navarro, Eduardo Fernández, Nicolás Garcia-Aracil

**Affiliations:** 1 Biomedical Neuroengineering Group, Universidad Miguel Hernández, Elche, Alicante, Spain; 2 Systems Engineering and Automation Department, Universidad Politécnica de Cartagena, Cartagena, Murcia, Spain; Politehnica University of Bucharest, ROMANIA

## Abstract

This paper presents the application of an Adaptive Resonance Theory (ART) based on neural networks combined with Fuzzy Logic systems to classify physiological reactions of subjects performing robot-assisted rehabilitation therapies. First, the theoretical background of a neuro-fuzzy classifier called S-dFasArt is presented. Then, the methodology and experimental protocols to perform a robot-assisted neurorehabilitation task are described. Our results show that the combination of the dynamic nature of S-dFasArt classifier with a supervisory module are very robust and suggest that this methodology could be very useful to take into account emotional states in robot-assisted environments and help to enhance and better understand human-robot interactions.

## Introduction

Recent developments in robotic technology have shown that robotic devices are able to play important roles in neurorehabilitation [1, 2], however there are still many challenges to be solved. As result, despite the increasing popularity of robots in neurorehabilitation, their effectiveness is still discussed controversially. One important issue in this field is to promote active patient participation in the loop control, which refers to the concept of acting cooperatively to the human instead of treating the human as a source of perturbation. Therefore an ideal robotic assisted device should be able to decide which level of difficulty should be applied in different rehabilitative scenarios taking into account biomechanical information as well as physiological and emotional aspects of the patients underlying robot-assisted therapies.

Emotion is a complex state of feeling which involves psychological and physiological reactions produced by the interactions between human being’s and the environment. A widely accepted classification of emotions, called also affective states, describe them as a circumplex with two dimensions: valence and arousal [3]. Valence can take values from displeasure state to pleasure state and on the other hand, arousal can take values from deactivation state (from sleep to drowsiness) to activation state (from various stages of alertness to frenetic excitement). The published studies of neural systems involved using neuroimaging techniques suggest that valence and arousal may be associated with separate neural circuits containing the amygdala, insula, thalamus, dorsal anterior cingulate cortex, and prefrontal regions [4–10]. Most of these studies show that the amygdala is the core of affective region suggesting that this region may belong to both valence and arousal neural systems.

The Network theory can be applied as well to the neural computation of emotion as it is described in Pessoa’s conceptual proposal of neural computations and emotion [11]. Based on this assumption, a Cognitive Regulated Affective Architecture (CRAA), which comprises a cognitive network, an affective network and an appraisal layer is proposed by Feng [12]. In addition, the affective network was designed to simulate the functions in amygdala and was built using a neural network based on Adaptive Resonance Theory (ART) models. For this reason, we hypothesized that Neural Networks, specially ART based neural networks, should work better than previous classifiers implemented to estimate the user’s emotional state based on physiological reactions in rehabilitation therapies assisted by robotic devices. An exhaustive list comparing the use of different classification algorithms accordingly with number of subjects enrolled in the studies, the classes used, the type of classifier with its accuracy and so on for psychophysiological studies were summarized and classified in Novak et al. [13].

In a previous work [14], we used nine machine learning techniques to estimate different user’s states such as bored-relaxed, pleased and excited-aroused. Our results showed that the Support vector machines (SVM) with Radial Basis Functions (RBF) kernels provided the best results in terms of accuracy (91.43%). However for a wider use of these technologies we need methods able to cover patients with a broad variety of physical as well as cognitive impairments and capable to adapt automatically to the patient’s specific demands and needs. Therefore, due to the widely use of neural networks in neural process modelling related with emotions, in this paper we have investigated the potential usefulness of neural networks incorporating concepts of fuzzy logic theory to estimate user’s emotional state and verify the performance of this technology.

## Materials and Methods

### Neuro-fuzzy Classifier: S-dFasArt

A neuro-fuzzy method of classification called S-dFasArt [15] (Supervised and Dynamic Fuzzy Adaptive System ART-based) has been used in this work to classify temporal patterns of a physiological signals set acquired during rehabilitation therapies assisted by a robotic device. This method combine the properties of neural networks based on Adaptive Resonance theory (more specifically is based on fuzzy ARTMAP architecture [16]), and the fundamentals of the Fuzzy Sets theory using a supervised-competitive learning and dynamic equations for the processing stages of the algorithm.

Its neuro-fuzzy architecture takes advantage of the learning capacity and adaptation of the neural network, and the robustness-interpretability of fuzzy systems. Moreover, the properties of the learning algorithm, the update mode and speed of convergence of the weights are improved. The proposed neuro-fuzzy architecture satisfies the stability-plasticity criterion since the classifier is able to maintain the accumulated knowledge and acquire new learning patterns and allows a quick learning with a small set of training patterns as well.

Due to competitive learning, all nodes or output categories react to an input value but the classifier only active the neuron with the highest response level. The category associated with the winner node is the classification of the network for the current input pattern.

#### S-dFasArt Architecture

**Fig 1:**
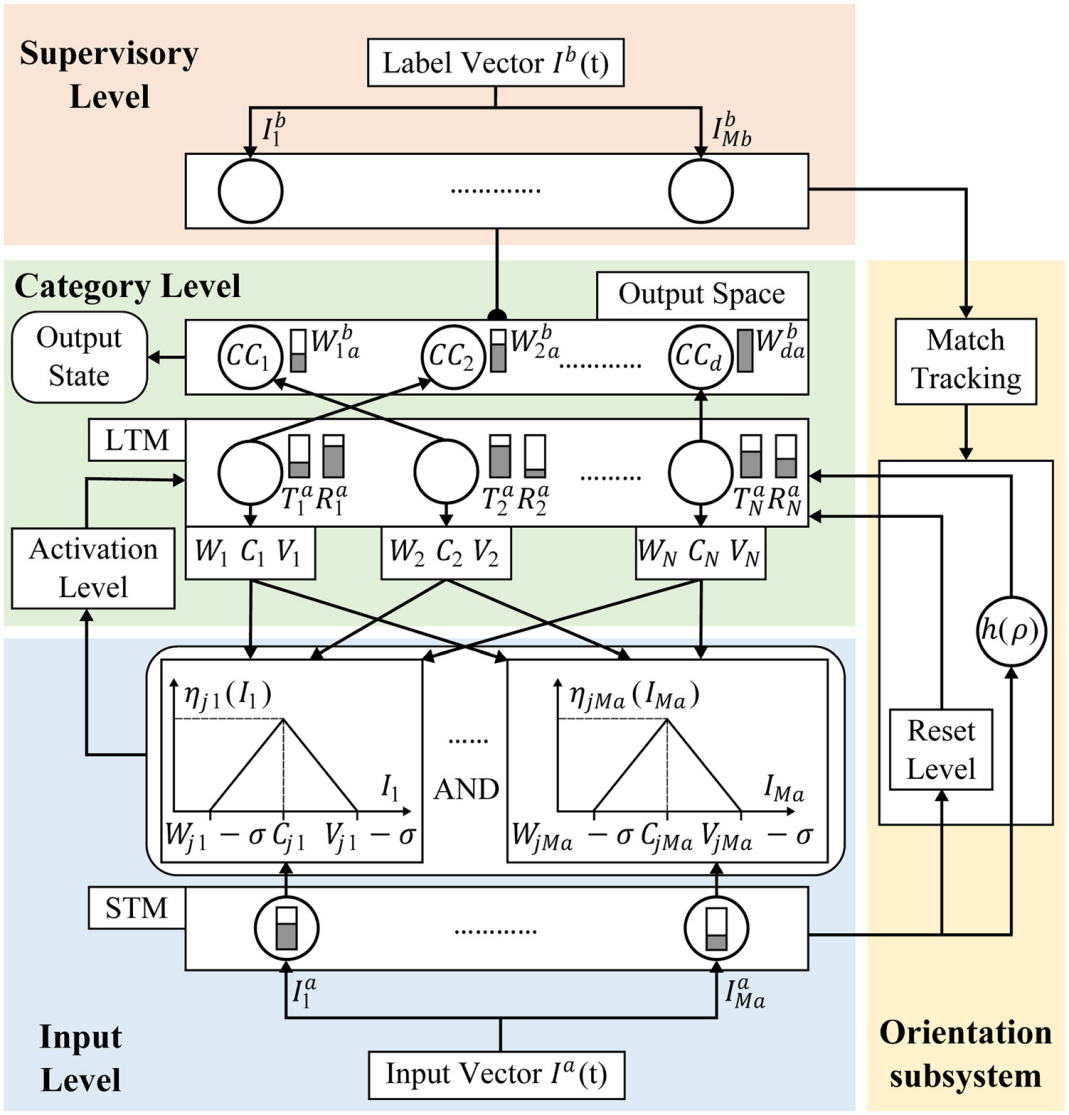
S-dFasArt Architecture.

Fig 1 shows the general architecture of the proposed classifier model, combining neural and fuzzy operations. This model consists of the following elements: an Input Level, a Supervisory Level, an Orientation Subsystem, and a Category Level.

The Input level is formed by *M*_*a*_ nodes. Each node receives one element of the *I* ^*a*^ vector to hold temporally the most important input aspects, activating the short-term memory STM. A fuzzy block integrated by a triangular fuzzy activation-membership function *η*_*ji*_ is associated to each of the input nodes. These blocks measure the membership degree of each input characteristics *i* regarding each fuzzy category *j*. The size of the membership function can be determined by the design parameter *σ*. So, this parameter modifies the diffuse character of the output categories [17].

The Supervisory Level has *M*_*b*_ nodes to present the pattern with the correct classification state *I* ^*b*^ associated to an input vector. This level configures the output space by creating a number *d* of classes with each of possible classifications that the network can encode. The input of the supervision vectors is only provided to the network during the learning stage.

The Category Level is formed by a set of *N* nodes representing all the categories that have been created during the learning process, resulting in a set of fuzzy units. Each node or category has two main associated values: *T*_*j*_ indicating the degree of activacion and *R*_*j*_ indicating the ability to learn from that input. These categories are activated with a determined level *T*_*j*_ when an input pattern is presented to the classifier. Three kinds of weights, minimun *W*_*ji*_, central *C*_*ji*_, and maximun *V*_*ji*_, are associated to each output unit. These weights store the long-term memory of the network, whose values are linked with the fuzzy blocks to update the membership functions that handle the acceptability level of the input pattern depending on the category that is reacting. The activation of output fuzzy categories is calculated using an AND operation of all the fuzzy degrees of membership of the input vector characteristics. Each fuzzy category can only encode one output state *CC* of the *d* possibilities that have been generated in the network during the learning phase from the supervision values. So different categories may point to the same classification node in the output space. Therefore, this level is responsible for linking the sequence pairs of input vectors with the supervision vectors.

There is also an Orientation Subsystem to detect the similarity of the input vector with the categories learned by the network. This similarity percentage can be compared with a vigilance parameter *h*(*ρ*) to control the number of diffuse categories that must be created in the Level Category. The vigilance parameter determines how strict the network must be in the classification process of the input measures, generalizing the results. The match tracking manages the network for the vigilance parameter is automatically set, indicating whether the entry was correctly classified in the node *j* or if the model has to create another output category. If the similarity calculated by the Orientation Subsystem is not sufficiently similar to a category, a reset level *Rj* is produced to disable current category and choose another category following the maximum similarity criterion.

#### Learning Algorithm

**Fig 2:**
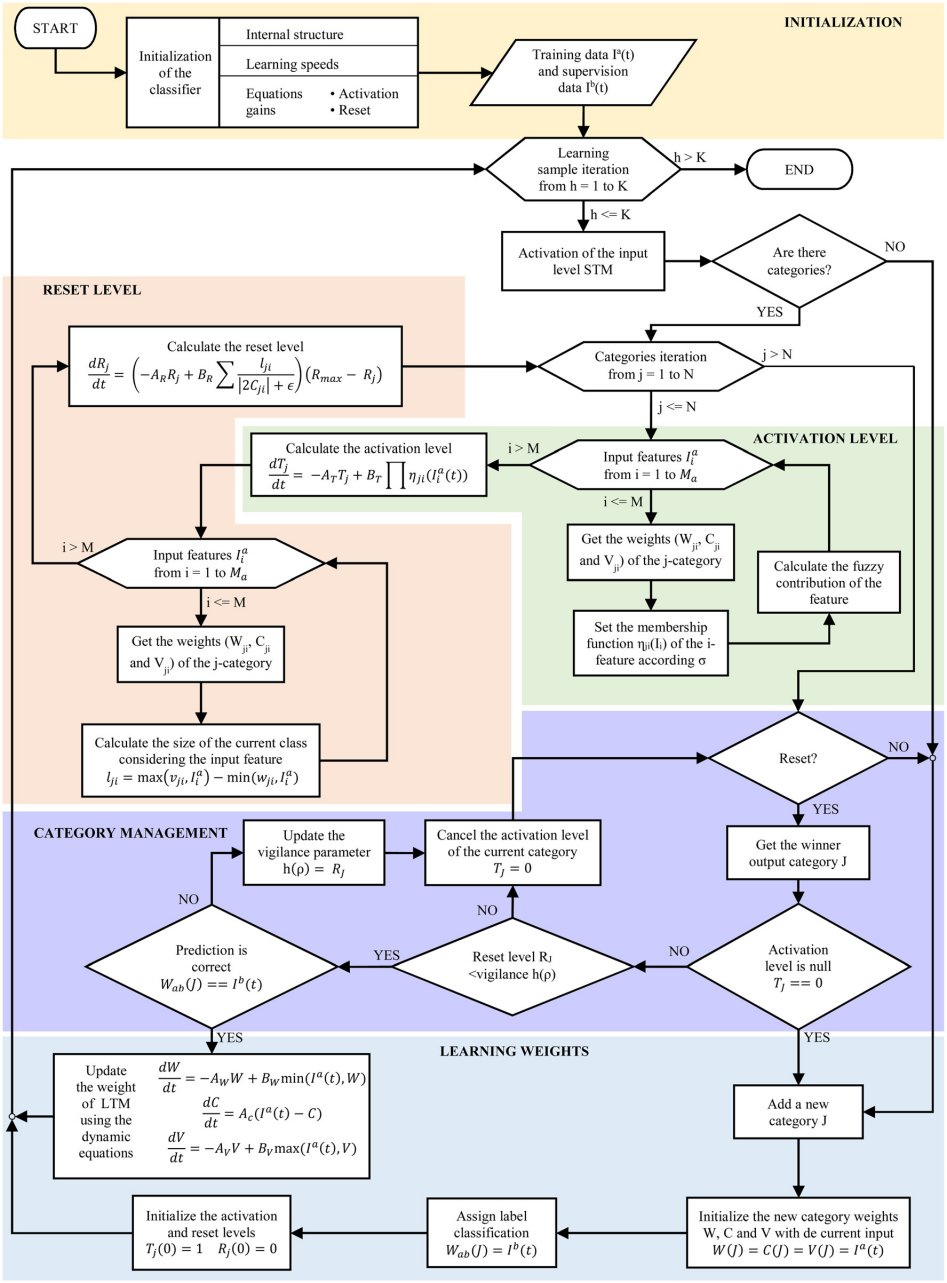
S-dFasArt Learning Algorithm.

In this section, the training algorithm implemented by the classifier during its learning stage is presented (Fig 2). A competitive learning is applied to generate new fuzzy categories, introducing the input data to the network only once, in the temporal order that these data are obtained and processed. With this process an update of the category weights is achieved. A more comprehensive description of the learning algorithm, its dynamics equations and the meaning of the network parameters, can be found in [18] and [19].

Nodes of the Category Level compete among themselves, but the learning phase only occurs in the neuron with the highest activation level. The S-dFasArt algorithm can be described with the following steps:

1. Firstly, the classifier is initialized, defining the values of the parameters of the dynamic equations, such as the activation rate, the growth rate of the level reset, time constants, linked gains or the vigilance parameter. These parameters affect the learning behavior directly. Initially, no input pattern has been presented to the network, so this network does not have any node associated with weights in the Level Category.
2. The training measures *I* ^*a*^(*t*) and its corresponding supervision labels *I* ^*b*^(*t*) assigned to this data are computed, getting K number of samples.
3. One input pattern *h* is presented to the network. Each node of the Input Level receives a feature of the input vector, activating the short-term memory to indicate the presence degree of each signal attributes. Also, the supervision vector is presented to the Supervisory Level, activating its nodes.
4. When the network gets an input pattern and a supervision label, checks whether there are categories to calculate their levels of activation and reset. If the network has no category, this step is not performed.
5. If there are categories, the following step sequence is executed for each of the existing *j*-categories:
  - The current category *j* sends the weights *W*_*ji*_, *C*_*ji*_ and *V*_*ji*_ to the fuzzy logic block associated with each of the *i*-nodes in the Input Level and updates its activation-membership function, considering the *σ* parameter to control the size and the diffuse character of the categories.
  - Then, the value of fuzzy membership associated with each input neuron that determine the activation level of each feature of the input vector is computed. The level activation *T*_*j*_ of the processed category can be calculated from these values and the AND operation, using the dynamic equation responsible of the activation process of the S-dFasArt algorithm (Eq 1). The parameter *A*_*T*_ is the activation speed of the categories, and determines the sensibility of the network to respond to input changes. $\eta_{ji}(I_{i}^{a})$ represents the fuzzy contribution of each feature of the input sample to compute the activation level, applying the activation-membership function. While the parameter *B*_*T*_ specifies the dynamic gain of the total fuzzy contribution.
    $$\begin{array}{c} \frac{{dT}_{j}}{dt}=-A_{T}T_{j}+B_{T}\prod_{i=1}^{M}\eta_{ji}(I_{i}^{a}(t)) \end{array}$$ (1)
  - Next, the size of the fuzzy category is calculated considering the input data as a pattern attached to this category, i.e. the similarity degree that indicates if the input pattern is a subset of the weights of the associated category. Therefore, each *i*-node of input provides a magnitude (Eq 2) to calculate the total size of the category as the sum of all these values (Eq 3).
    $$\begin{array}{c} l_{ji}=max(V_{ji},I_{i}^{a})-min(W_{ji},I_{i}^{a}) \end{array}$$ (2)
    $$\begin{array}{c} dreset\;=\sum_{i=1}^{M}\frac{l_{ji}}{|2C_{ji}|+\epsilon } \end{array}$$ (3)
  - This size is used to calculate the level reset of the current category from the dynamic equation of S-dFasArt reset (Eq 4). *R*_*j*_ can be considered as a similarity threshold required for an input vector can be associated with the category *j*. *A*_*R*_ involves the growing speed of the Reset Level that allows the system to be able to respond to the dynamic changes of the input data. This implies the aptitude of the category to learn from the input that is activating it. The *B*_*R*_ parameter determines the gain linked to the total size of the category. *R*_*max*_ denotes the maximum Reset value for the category.
    $$\begin{array}{c} \frac{{dR}_{j}}{dt}=({-A}_{R}R_{j}+B_{R}dreset)(R_{max}-R_{j}) \end{array}$$ (4)
6. At this point, the levels of activation and reset of the *N* categories that currently exist in the network have been calculated. Then, the winner category *J* is determined selecting the category with the highest activation value (Eq 5).
  $$\begin{array}{c} T_{J}=max\{T_{j};\;j=1...N\} \end{array}$$ (5)
7. If the maximum activation level is null (*T*_*j*_ = 0), an uncommitted category is added. To establish the weights of this uncommitted node as a prototype of the input pattern, a fast commit is applied (Eq 6). Then, the label received in the Supervisory Level is assigned as classification result of the current category (Eq 7) in the output space. Also, the levels of the activation and reset at the first instant of time are initialized (Eq 8).
  $$\begin{array}{c} W=C=V=I^{a}(t) \end{array}$$ (6)
  $$\begin{array}{c} W_{ab}=I^{b}(t) \end{array}$$ (7)
  $$\begin{array}{c} T_{j}(0)=1\;\;R_{j}(0)=0 \end{array}$$ (8)
8. However, if the winner category have a no null activation level, its reset level is compared with the vigilance parameter. If the reset value exceeds the vigilance threshold, a reset state occurs because the winner neuron does not properly represent the category in which the current input pattern belongs. Then, the Orientation Subsystem temporarily disables the node *J* (*T*_*J*_ = 0) and selects the category whose level activation is the next highest.
9. If the reset level is less than the vigilance parameter, the classification label of the winner category is compared with the data *I* ^*b*^(*t*) received in the Supervisory Level. If these values do not match, the prediction is incorrect, consequently the vigilance parameter is automatically set using the value of the current reset level to search a new category, canceling its activation level *T*_*J*_ = 0.
10. If the classification state of the winner neuron is the same as the label of the Supervisory Level, the activated node is the category most appropriate for the current learning sample *I* ^*a*^(*t*). Thus, an updating process of the weights *W*_*ji*_, *C*_*ji*_ and *V*_*ji*_ is applied in the selected category using a slow recode to increase its resemblance to the input data. This update of the weights is performed from the dynamic equations of S-dFasArt learning (Eq 9). The parameters *A*_*W*_, *A*_*C*_, *A*_*V*_, *B*_*W*_ and *B*_*V*_ can be interpreted as learning speeds associated to the growing of the minimun, central and maximum weights respectively of the fuzzy categories.
  $$\begin{array}{ccc} \frac{dW}{dt} & = & -A_{W}W+B_{W}min(I^{a}\left(t\right),W)\\ \frac{dC}{dt} & = & A_{C}(I^{a}\left(t\right)-C)\\ \frac{dV}{dt} & = & -A_{V}V+B_{V}max(I^{a}\left(t\right),V) \end{array}$$ (9)

### Methodology and Experiments

**Fig 3:**
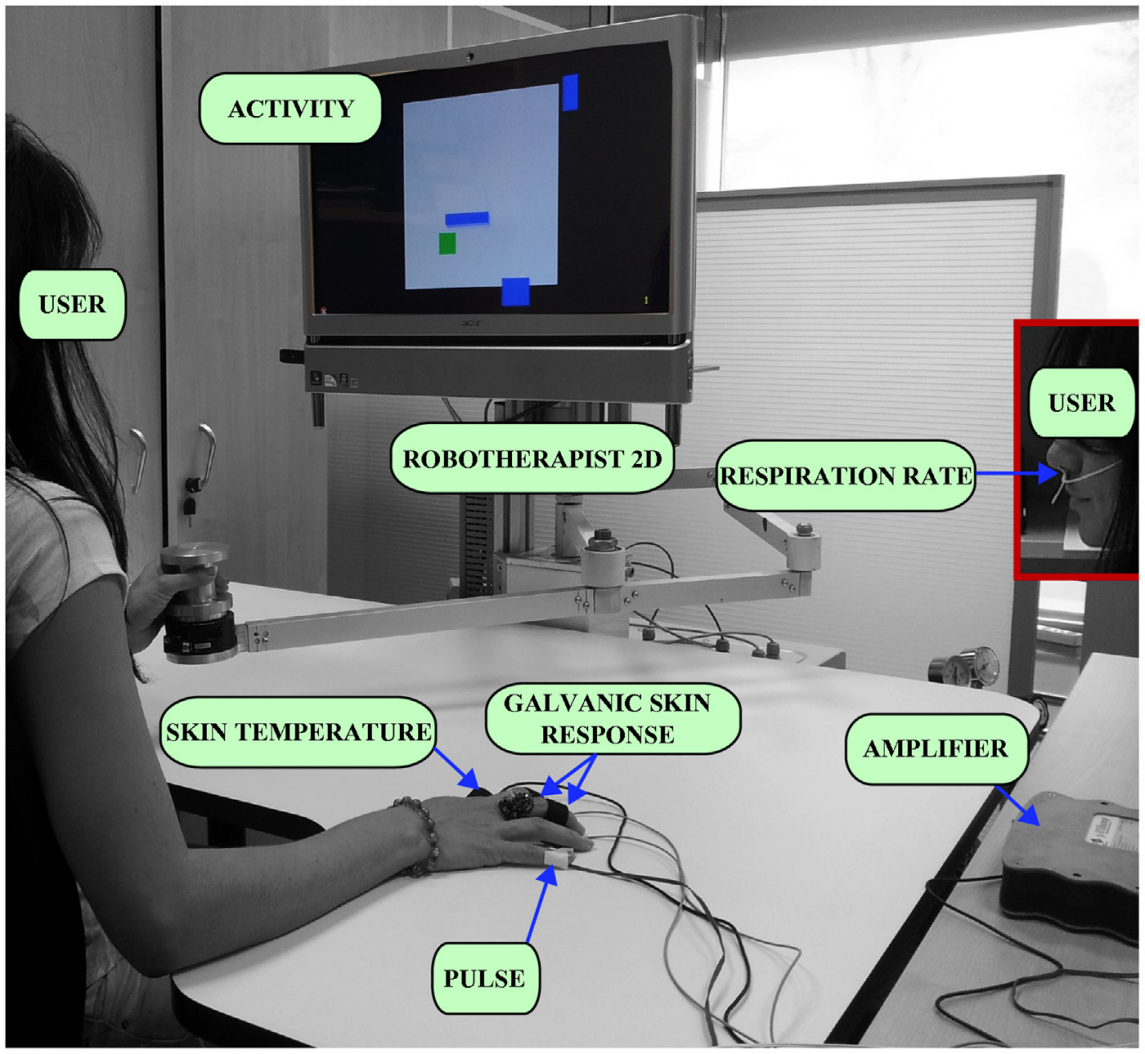
One subject during the experiments.

The experiments were conducted as we have previously reported [14]. Briefly, a hardware configuration composed by a robotic device, a signal acquisition system and a virtual reality system were used to perform the requested activities and to monitor in real time the user’s physiological signals (pulse rate, respiration rate, skin conductance level (SCL), skin conductance response (SCR) and skin temperature). The robotic device used for these experiments was the PUPArm robot system, a commercial platform designed for upper-limb assisted therapy which is now commercialised by Instead Technologies Inc with the trading name of “RoboTherapist 2D” (see Fig 3).

A specific activity to induce different user’s psychophysiological states was designed. The activity consisted of three main components: the area of activity, bounded by a black frame, the pointer that moves the user represented by a green square and a series of blue rectangles of different sizes moving randomly across the screen. The goal of the activity was to move freely around the screen avoiding the collision with the blue rectangles without leaving the space delimited by the black frame. Every time the subject touches a blue box or leaves the area of activity means a mistake what implies that the user square turns red and sounds a shrill sound. Three levels of difficulty: relax, medium and stress level were defined depending on the number of blue rectangles and their speed.

**Fig 4:**
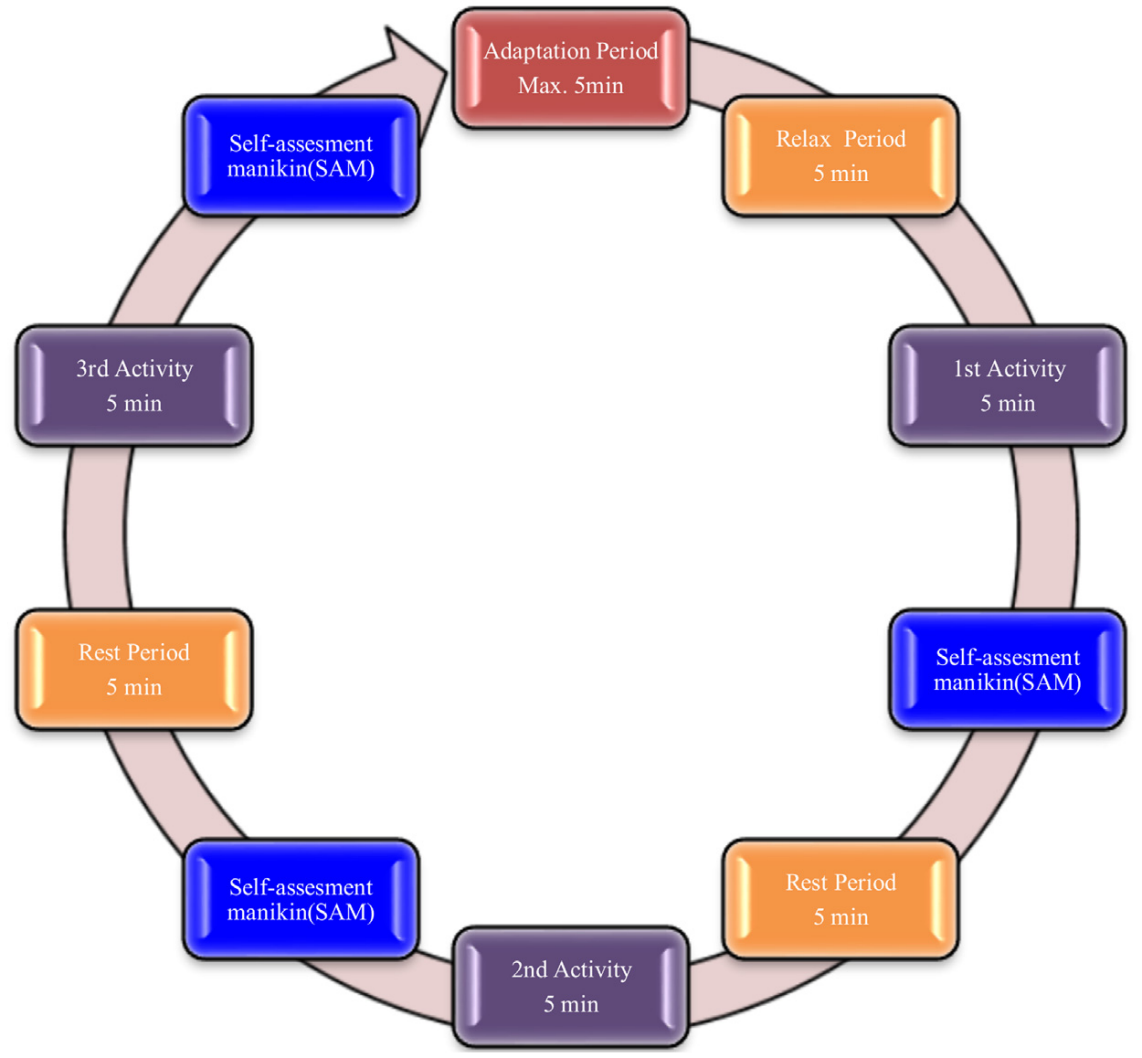
Experiment protocol. This protocol is composed of three activities and rest periods before and after activities.

Human data presented in this article have been acquired under an experimental protocol approved by the Medical Ethics Committee of the Universidad Miguel Hernandez of Spain and all subjects gave written informed consent. Seven volunteers participated in the experiments. All were healthy, without cognitive or physical deficits. They were aged between 26 and 42 (mean age 31 years, median age 29 years, standard deviation 6.3 years). The whole experimental protocol is shown in Fig 4. After each activity, a self-assessment manikin (SAM) was presented to the subjects to measure their affective responses. The dataset used in this experimentation is provided in S1 Dataset.

Once the physiological signals were acquired, a normalization of the features was completed as we have previously reported [14]. After that, a Principal Components Analysis (PCA) was used in order to study the possibility of further reducing the number of input features for the machine learning algorithms and the proposed neuro-fuzzy model based on the S-dFasArt architecture.

## Results

A test classification was performed to check the possibilities of the proposed neuro-fuzzy model based on the S-dFasArt architecture, using the cross-validation technique called Leave-one-Out (LOOCV). The network generalization in situations where the network was not trained was examined with this validation method, estimating the performance of the classifier. This technique is suitable when the experimental data do not contain sufficient measurements.

An adjustment process of the S-dFasArt classifier should be applied to obtain a model of functional classification, presenting a set of learning samples to the algorithm explained in Materials and Methods section. The learning information data has been computed by acquiring and processing the physiological responses of seven subjects.

The adjustment of the classifier basically can be divided into three phases. First, the parameters linked to the dynamic equations of S-dFasArt are initialized with default values. Secondly, a learning of the weights that represent the fuzzy categories is effected. Finally, a phase of parameter adjustment is applied to calculate the two network parameters that have more influence in the classification data, and get the better interpretation values. These parameters are related to diffuse character *σ* of the categories and their activation speed *A*_*T*_. However, the parameter *A*_*T*_ is not adjusted in this work and its default value is maintained due to the nature of the validation method, where one sample is only tested in each iteration, therefore a continuous calculation of the categories activations is not required.

Before starting the training of the network, the parameter *A*_*R*_ is set. This parameter controls the number of categories that the network generates. First, a quantitative study of the categories that are generated based on *A*_*R*_ is performed to establish a range of values which allow a reasonable generation of categories for the amount of learning samples presented to the network. Thus, the creation of categories is limited, avoiding an excessive generation due to an overtraining caused by the categories proliferation problem that S-dFasArt inherits from the ARTMAP architecture and providing a generalization of the categories.

**Fig 5:**
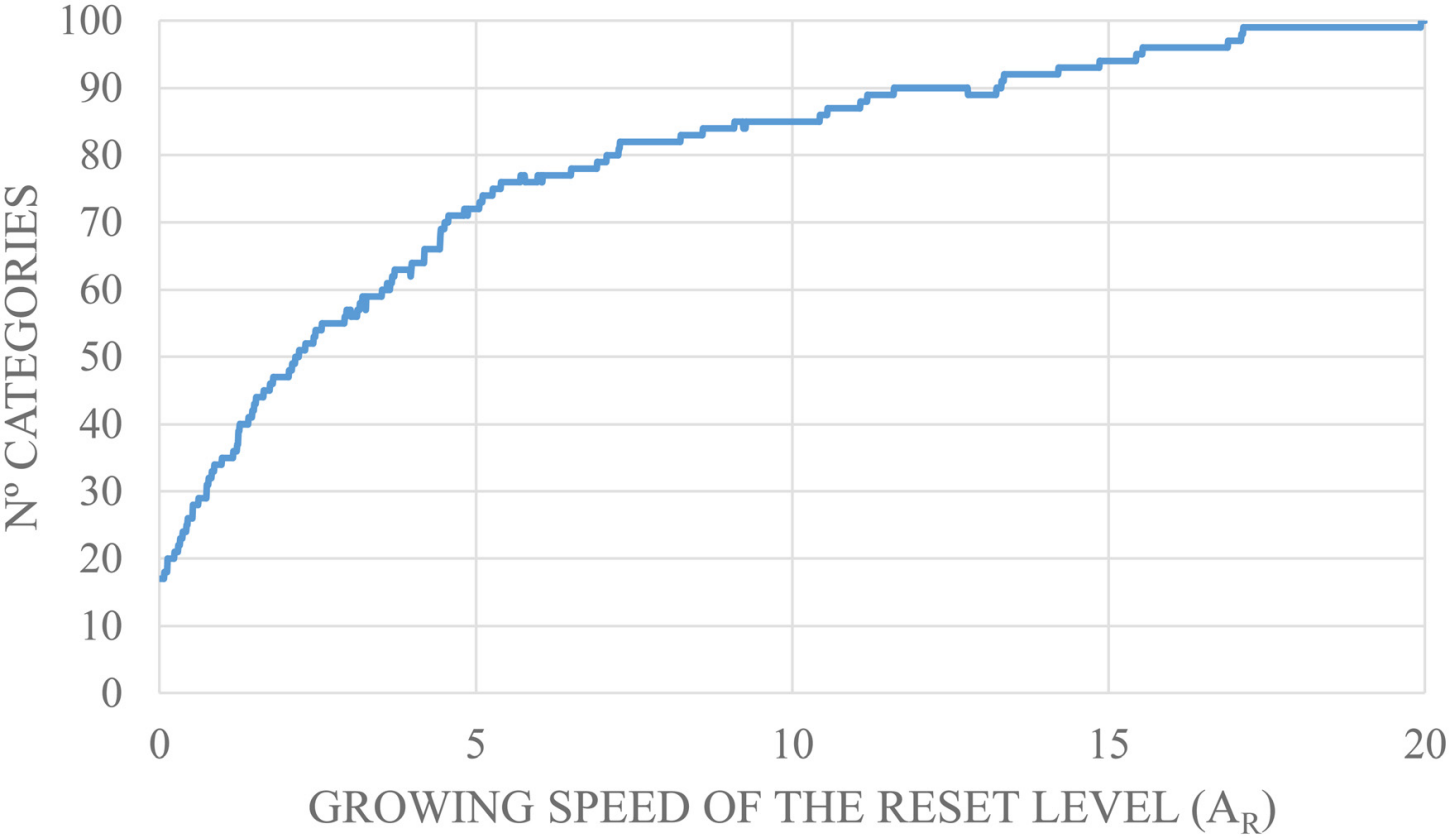
Proliferation of categories.

Then, the values of the parameter *A*_*R*_ is established between a range from 0 to 20 in order to compute the categories that the S-dFasArt architecture can generate using this data type. The number of committed categories (nodes) is not prefixed beforehand in the models based in ARTMAP. This value depends of the learning processing. In S-dFasArt, the categories are generated depending on the number of learning samples which are supplied to the model. One classification model is implemented for each value of *A*_*R*_ applying default values of network and all the learning samples. After that, the number of categories for all models is computed. Fig 5 shows the generation in the fuzzy categories depending on the growing speed value *A*_*R*_ of the reset level.

An increase in the amount of categories was observed in the graph, due to the growth of the *A*_*R*_ value. Therefore, *A*_*R*_ values that achieve a number of categories between a range from 20 to 40 were selected to avoid the generation of too many categories which could cause the network to incorrectly classifies the samples. The range of generated categories is establised with these values because the dataset had 105 samples, 15 per user. Then, the *A*_*R*_ value was set between 0.05 and 2.15. Finally the Leave-one-out technique was used to validate the S-dFasArt classifier.

With this validation technique, K-classification models are generated according to the amount of learning measures that have been extracted from the physiological signals. In each iteration, a classification model is created using all the learning samples with the exception of one measure that is removed from the set. These samples are classified with the trained network during the test phase. The remaining data set is used in the weights learning phase during the adjustment process of the classifier. This procedure is repeated one time for each measure, then the classification success percentage of the network is computed using the arithmetic mean of the iterative process.

Now, the value set of parameters *A*_*R*_ and *σ* that offers better results is calculated using the trial and error method. Since the range of G-values of the parameter *A*_*R*_ is already configured, *σ* is established with a range of L-values between 0.0001 and 1.0, so this parameter has values that cover a wide range of sizes of the triangular fuzzy activation-membership function. In this way, the validation method Leave-one-out is performed G*L times, depending on the range value of the *A*_*R*_-*σ* data set. To conclude, the result with the highest success rate provides the best values for *A*_*R*_-*σ* data set.

The remaining parameters are kept constants. The reason for this is that its default values provide the generation of models with the best classification rates. These values are also obtained through trial and error. Table 1 shows the values applied during the process of adjustment and validation of the classifier, together with a brief explanation for each parameter.

**Table 1:** S-dFasArt network parameters.

**PARAMETER**	**VALUE**	**DESCRIPTION**
*A*_*R*_	0.05–2.15	Growing speed of the reset level
*σ*	0.0001–1.0	Diffuse character of the fuzzy categories
*A*_*T*_	0.01	Activation speed of the fuzzy categories
*A*_*W*_	0.8	Growing speed of the weights associated to the fuzzy categories
*A*_*C*_	0.8	
*A*_*V*_	0.1	
*ε*	0.001	The minimum value of the diffuse character
*α*	1e-30	Activation value to generate new categories
*h*(*ρ*)	0.1	Vigilance parameter
*R*_*max*_	0.2	The maximum reset value to disable the fuzzy categories
*B*_*T*_, *B*_*R*_	1	Gains of the S-dFasArt differential equations
*B*_*W*_, *B*_*V*_		

Values and descriptions of the S-dFasArt network parameters.

**Fig 6:**
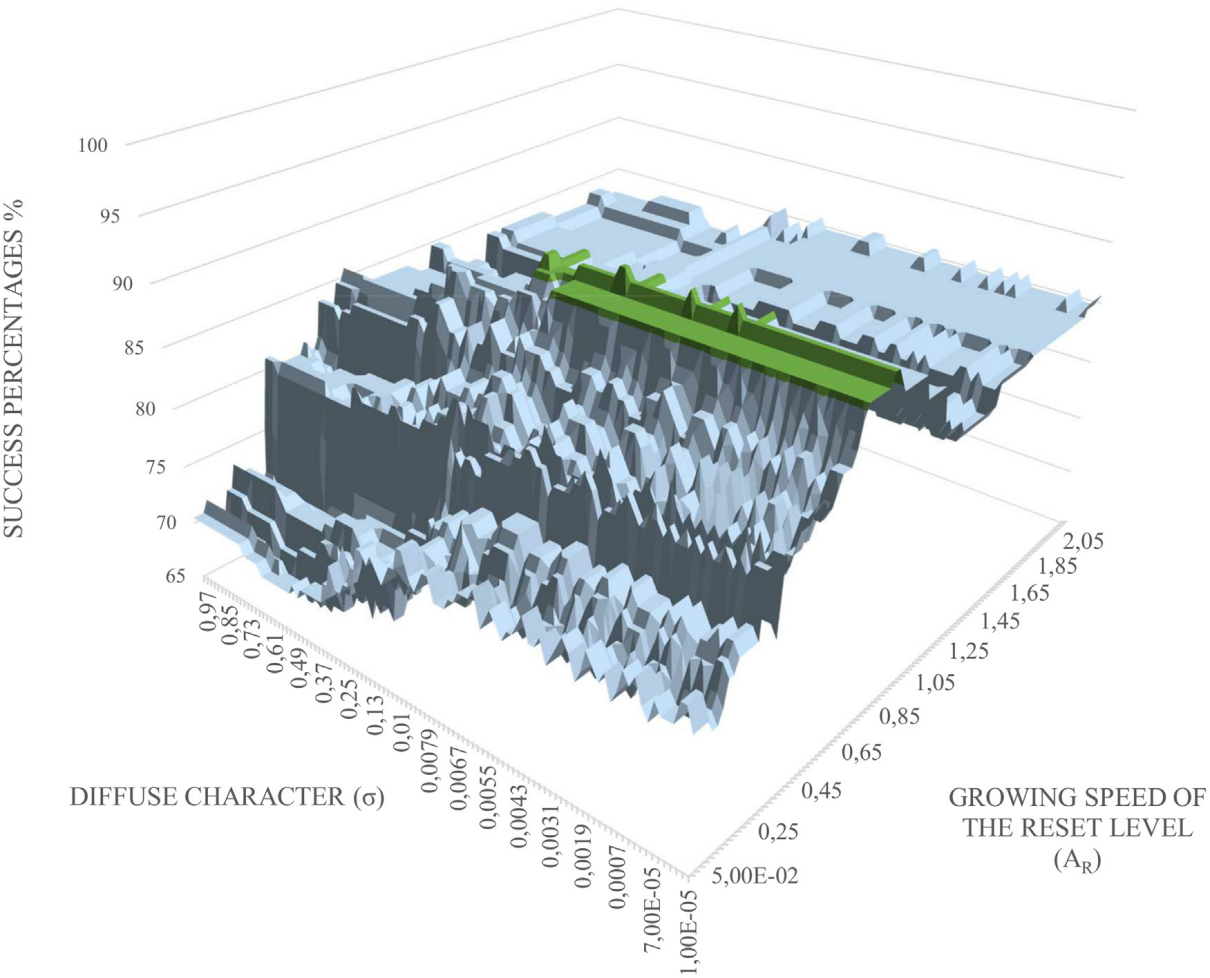
Success percentages depending on *A*_*R*_ and *σ*.

**Fig 7:**
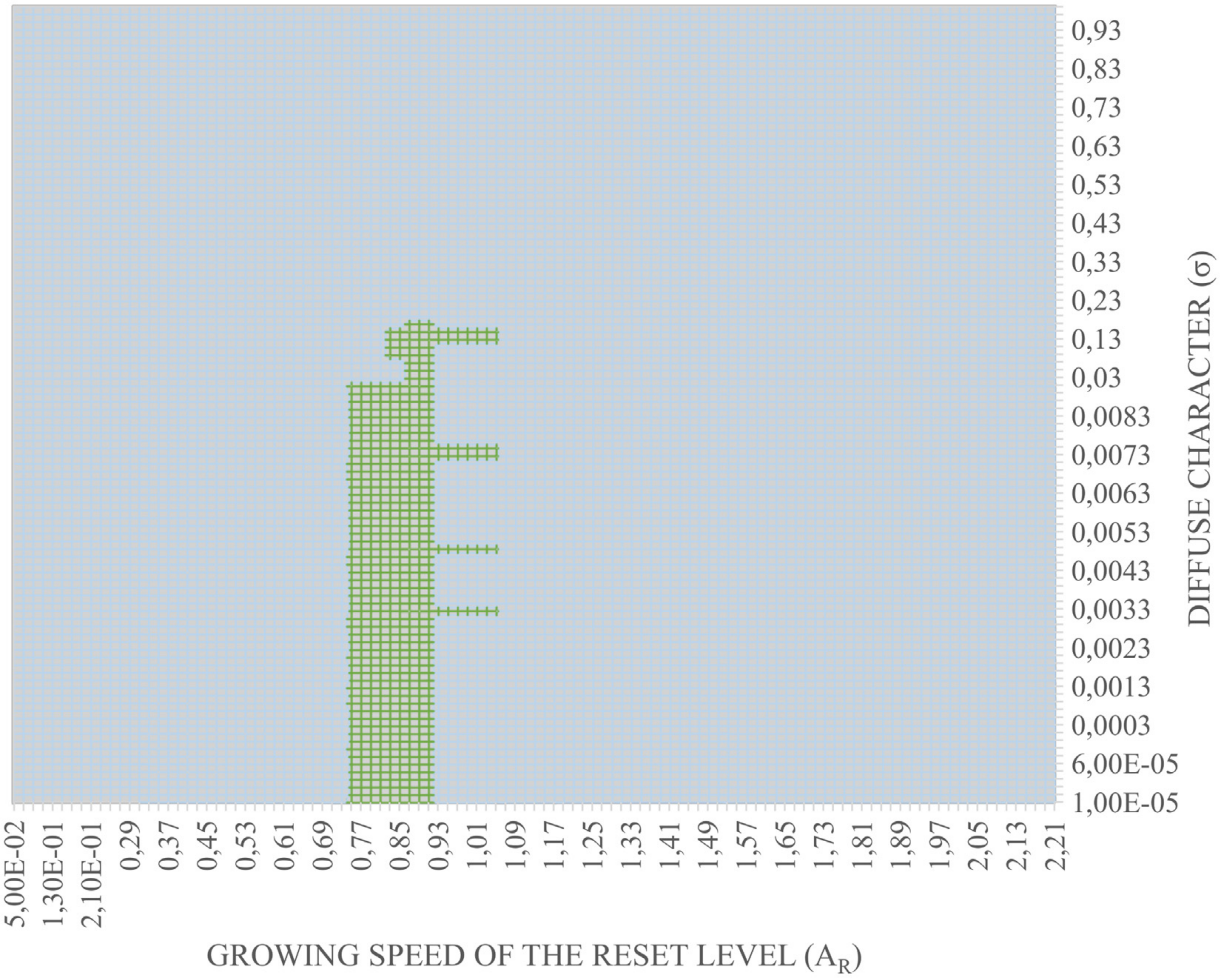
*A*_*R*_ and *σ* values plane.

Once all classification models are processed, and the sets of *A*_*R*_ and *σ* parameter values are tested, 11130 possible results have been computed. Fig 6 shows a 3D graph that collected the values of the success rates of all generated classification models. The highlighted zone of the graph are the points whose success percentages are greater than 90%. Then, Fig 7 shows a 2D plot of the *A*_*R*_ and *σ* values for a success percentage greater than 90%.

However, to get the highest success percentage, the selected values are the ones in the maximum peak of the graph. At this point, the third phase of the adjustment process of the classifier is accomplished, getting the set of the parameters *A*_*R*_ and *σ*, whose values provide the better success rate. Final results during the development of this experiment are summarized in Table 2. From the 11130 possible computed models, five classification models that obtained the best results in terms of success rate, have been selected. The first column shows the categories generated by the iterations of the validation method. The next two columns recollects the values of the adjusted parameters obtained in the adjustment phase. The *A*_*R*_ parameter affects directly in the value of the generated categories. The success rates have been placed in the last column. The variability of the success rates related to *A*_*R*_ and *σ* can be observed in this Table.

**Table 2:** Summary results table.

Categories	*A*_*R*_	*σ*	Success %
33–34	0.87	0.0033	92.38
33–34	0.89	0.0083	91.43
30–31	0.75	0.01	90.48
34–35	0.99	0.17	89.52
28–29	0.69	0.05	87.62

The best success results.

The classification ranges of the S-dFasArt model compared with some of the classifiers [14] used to interpret this signal type have been collected in Table 3. The LOOCV results is presented in six column table to show the classification values applying Principal Components Analysis to the input data and the results without employ any complementary processing. The performance of each of the nine machine learning and the proposed neuro-fuzzy model using different number of principal components (PC) as input data, are presented in the first five columns, while the data without PCA computation can be shown in the last column.

**Table 3:** Comparison of classification methods.

**ALGORITHM**	**PCA 1 PC**	**PCA 2 PC**	**PCA 3 PC**	**PCA 4 PC**	**PCA 5 PC**	**NO PCA**
**PLA**	56.57	81.9	83.5	82.86	82.1	83.05
**LR**	61.90	65.71	84.76	83.81	85.71	85.71
**LDA**	50.48	64.76	74.29	74.29	76.19	76.19
**QDA**	49.52	63.81	75.24	78.10	78.10	78.10
**SVM**	60.00	67.62	86.67	85.71	85.71	85.71
**SVM with RBF**	78.10	80.00	91.43	91.43	91.43	91.43
**NB**	49.52	61.90	66.67	64.76	60.00	53.33
**KNN**	64.76	72.38	80.95	80.95	80.95	80.95
**RBF**	58.10	57.05	56.10	56.10	56.29	56.19
**S-dFasArt**	69.52	80.95	90.48	90.48	90.48	92.38

Results of Leave-one-out cross-validation (LOOCV).

## Discussion

The hypothesis of the present work is based on the Pessoa’s conceptual proposal of neural computations and emotion. Based on this assumption, a CRAA, which comprises a cognitive network, an affective network and an appraisal layer, was proposed by Feng. Moreover, that affective network of CRAA was developed using a neural network based on ART models. This point was one of the pillars that support our hypothesis: “*ART based neural networks should work better than classifiers implemented on previous works since they are widely used to model neural process related with emotions*”.

The results of the application of an Adaptive Resonance Theory (ART) based neural network combined with Fuzzy Logic systems, which is known as S-dFasArt, in order to classify user physiological reactions performing robot-assisted rehabilitation therapies are presented and compared with the results of the application of nine machine learning techniques. The S-dFasArt approach obtained better results in terms of accuracy (92.38% in LOOCV) than the SVM with RBF kernel model with 91.43% in LOOCV (See Table 3). These results show that the combination of the dynamic nature of dFasArt with a supervisory module produces a robust classifier capable to provide very good results despite of a small set of input data.

The proposed algorithm has been applied to problems associated with the classification of time-varying signals with high noise contamination or the classification of vehicle handling using GPS [18] data and electroencephalogram signals [15]. The signals of these type of application are very noisy and their temporary arrangements are a relevant feature to be studied. Since bio-signals used in user’s emotional state based on physiological reactions are very noisy, this architecture has been tested.

S-dFasArt allows an adaptation between its complexity and the dataset used in its learning step. The complexity (categories number) increases depending of the variability of the learning data. This fact produces that initial premises have not to be assumed. The larger the dataset is, more categories are generated by the model, resulting in a more robust system. Furthermore, this algorithm is robust against the contradictory and inconsistent data, which produce serious problems in the optimization mechanisms of other types of classification models.

Also, in Table 3, the performance of the S-dFasArt when applying PCA analysis can be seen. These results indicate that the feature reduction processing of the input data did not provide any improvement. Therefore, this method have to be used without PCA processing. Moreover, it seems that the S-dFasArt would have better results with a large input data set, and it will be more robust to noisy input data than SVM with RBF approach. If this assumption is corroborated, the presented approach can be considered as the best candidate to classify user physiological reactions in robot-assisted rehabilitation therapies.

There are several reasons to use this type of classification technique to estimate the user’s emotional state. One of this reasons is its performance against noisy data. Another one is its capacity of automatic adjustment to update the classification models and the networks parameters in real time. This way, the classification method can be adapted automatically to the patient’s specific demands and needs. This architecture can take advantage to classify this type of signals because it is able to allow the acquisition of new learning examples without deleting the accumulated knowledge.

## Supporting Information

S1 Dataset Dataset of physiological signals. The dataset contains temporal patterns of physiological signals acquired during robot-aided rehabilitation therapies. The first column reports the number of user. The main features of these physiological signals are registered in columns 2–6. The vector produced by these five values, represents the input pattern that indicate the presence degree of each signal attributes. The input pattern is ordered as follows: Pulse Rate, Skin Conductance Level (SCL), Skin Conductance Response (SCR), Respiration Rate and Skin Temperature. The last two columns include the supervision label of each input pattern and its corresponding difficulty level.
(CSV)
